# Statin therapy in the treatment of active cancer: A systematic review and meta-analysis of randomized controlled trials

**DOI:** 10.1371/journal.pone.0209486

**Published:** 2018-12-20

**Authors:** Mohammed A. M. Farooqi, Nikita Malhotra, Som D. Mukherjee, Stephanie Sanger, Sukhbinder K. Dhesy-Thind, Peter Ellis, Darryl P. Leong

**Affiliations:** 1 Department of Medicine, McMaster University, Hamilton, Canada; 2 Population Health Research Institute, McMaster University and Hamilton Health Sciences, Hamilton, Canada; 3 Department of Oncology, Division of Medical Oncology, McMaster University, Hamilton, Canada; 4 Health Sciences Library, McMaster University, Hamilton, Canada; Laurentian University, CANADA

## Abstract

**Background:**

Preclinical evidence suggests statins may have anti-tumor properties. Large observational studies are also consistent with improved survival and cancer-specific outcomes among cancer patients on statins. We sought to evaluate the randomized controlled trials of statins in addition to usual anti-cancer therapy.

**Methods:**

A systematic search of MEDLINE, Embase, CINAHL, Cochrane Library, Web of Science, Papers First and Clinicaltrials.gov was performed from inception through to July 4, 2017 to identify randomized clinical trials that investigated statin therapy in cancer patients. Our primary outcome was overall survival and our secondary outcome was progression-free survival. We calculated summary hazard ratio’s (HR) and 95% confidence intervals (CI) based on random-effects models using aggregate data. PROSPERO (CRD42017065503).

**Results:**

Ten studies with 1,881 individuals were included with 1,572 deaths and a median follow-up of 23 months. All trials included patients with advanced (stage 3 or higher) disease. There was minimal between-study statistical heterogeneity (*I*^*2*^ = 1.8%, for OS; *I*^*2*^ = 0%, for PFS). The pooled HR for overall survival in patients randomized to statins plus standard anti-cancer therapy versus standard therapy alone was 0.94 (95% CI, 0.85 to 1.04). In the 9 studies that reported progression-free survival (1,798 participants), the pooled HR for statin plus standard therapy versus standard therapy alone was 0.97 (95% CI, 0.87 to 1.07).

**Conclusions:**

In patients with advanced cancer and a prognosis <2 years, the addition of statins to standard anti-cancer therapy does not appear to improve overall survival or progression-free survival. Future research should assess if cancer patients with better prognosis benefit from longer-term statin therapy.

## Introduction

There is a strong experimental rationale to suggest statins may improve cancer-specific outcomes. Preclinical evidence indicates statins inhibit tumor growth and induce apoptosis in a number of tumor types [1–4]. Statins inhibit 3-hydroxy-3-methyl-glutaryl-coenzyme A (HMG-CoA) reductase, which is the rate limiting step in the mevalonate pathway. Mevalonate is a precursor of geranylpyrophosphate and farnesylpyrophosphate, products that regulate intracellular G-proteins Ras and Rho, which in turn regulate signal transduction of membrane receptors crucial for the transcription of genes involved in cell proliferation, differentiation, angiogenesis and apoptosis [5, 6]. Statins have also been studied for their potential chemo-sensitising effects through impairment of the Ras family signalling [7]. Consistent with the *in vitro* effects of statins, their efficacy has also been demonstrated in animal models of breast, colon, lung and hematological cancers [8–11].

Observational data from large prospective cohort and registry studies suggest statin use improves survival in patients with cancer [12]. A meta-analysis of 1,111,407 cancer patients with a variety of cancers at all stages showed that statin use was associated with a 30% reduction in all-cause mortality and a 40% reduction in cancer-specific mortality [13]. Reductions in all-cause mortality with statins may be explained by their theoretical anti-cancer effects, but may also be explained by potential cardiovascular benefits, as statins have shown improvements in cardiovascular mortality in both high- and intermediate-cardiovascular risk patients [14, 15]. However, these observational data may be subject to significant biases.

The aim of this study was to evaluate whether, in randomized, controlled trials, statins reduce all-cause mortality and improve cancer progression-free survival in patients with an active cancer. We performed a systematic review and meta-analysis of randomized controlled trials of statin therapy in cancer patients that report cancer-specific outcomes and overall survival.

## Methods

### Data sources and searches

A systematic literature search of MEDLINE, Embase, CINAHL, Cochrane Library, Web of Science, Papers First and Clinicaltrials.gov was performed from inception through to July 4, 2017 to identify randomized clinical trials that investigated statin use in cancer patients. We also conducted a manual screen of references cited in the retrieved articles. Unpublished studies were not included. The search strategy used the Medical Subject Heading and text key words *HMG-CoA reductase inhibitor*, *HMGCoA RI*, *statin*, *pravastatin*, *simvastatin*, *lovastatin*, *atorvastatin*, *cerivastatin*, *rosuvastatin*, *fluvastatin*, and *cancer*. We have attached our full literature search strategy as a supplementary file (S1 File). The literature search was conducted by one author (S.S.) and the search strings reviewed by two authors (M.F. and D.L.). This review was registered on PROSPERO (CRD42017065503).

### Study selection

Studies were eligible if they met all the following criteria: 1) randomized clinical trial, 2) statin used as intervention, 3) only patients with active cancer studied, 4) survival and/or cancer-specific outcomes reported. We excluded trials without a control arm to minimize the risk of bias from unmeasured confounders. Animal studies were not included. We limited inclusion to full manuscripts and did not limit by language. All potentially relevant abstracts were independently reviewed by two authors (M.F. and N.M.). Discrepancies were resolved by discussion with the senior author (D.L.). Papers identified after title and abstract screening were obtained in full.

### Study endpoints

The primary endpoint was overall survival (OS), measured from the date of randomisation until death from any cause. The secondary end-point was progression-free survival (PFS), measured from the date of randomisation to progression or death from any cause.

### Data extraction and quality assessment

Data were independently extracted from eligible articles by two authors (M.F. and N.M.). We used a standardised data abstraction form to collect the following descriptive information: study design, country where the study was performed, cancer type, cancer stage, treatment arms, sample size, follow-up period, number of outcome events and hazard ratio (HR)’s with corresponding 95% confidence intervals (CI). If the hazard ratio and corresponding 95% CI were not available [16–21], they were estimated using Kaplan–Meier (KM) curves as described by Tierney et al. [22] and Parmar et al. [23]. Briefly, this method involves digitizing the published KM curves to extract survival probabilities at particular time intervals, thus estimating numbers of events and numbers at risk. The time intervals were selected such that the event rate within each interval was no more than 20% of those at the start of the time interval. This approach assumes uniform censoring over time. The methodological quality of the studies was assessed using the Cochrane Collaboration’s risk of bias assessment tool [24]. A summary risk of bias table is included as a supplementary file (S1 Table).

### Data synthesis and analysis

We conducted analyses using STATA (version 15; Stata Corporation, College Station, TX, USA). Pooled HR’s for OS and PFS were calculated using a DerSimonian and Laird random effects model [25]. Between-study heterogeneity was determined using the *I*^*2*^ statistic, with a value >50% indicating increased heterogeneity. Several methods were used to assess the potential for publication bias. Visual inspection of funnel plots for both overall survival and progression-free survival were conducted [26]. Begg’s rank correlation and Egger’s weighted regression method were also used for both outcomes[27, 28].

## Results

### Study selection

**Fig 1:**
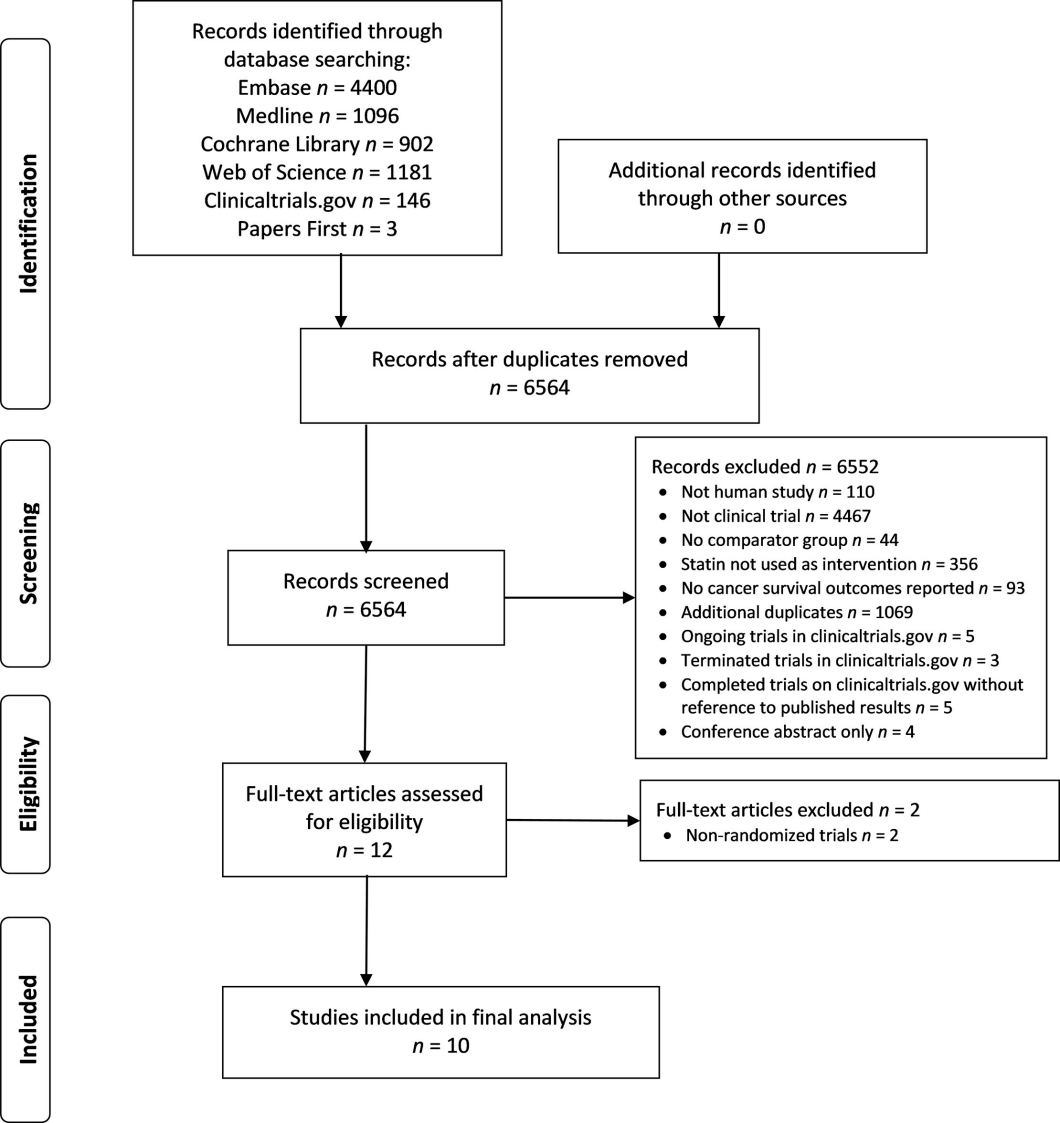
Flow diagram of the study selection as per the PRISMA statement.

Our initial search yielded 5495 unique potential citations (Fig 1). Of these, 5479 were excluded after reviewing titles and abstracts. Out of the trials that met screening criteria on Clinicaltrials.gov, 5 are ongoing (NCT02497638, NCT01441349, NCT01156545, NCT02958852 and NCT03024684); 3 are listed as terminated without any published results (NCT01011478, NCT02819869, NCT01342887); 5 are listed as complete without a reference to published results (NCT01759836, NCT01075555, NCT01903694, NCT01357486, NCT01418729). The authors of the completed trials have been contacted for references and only one has responded (NCT01357486)–this trial is due to be presented at the 2018 EASL (European Association for the Study of the Liver) conference. An attempt was made to obtain the full texts of the 16 remaining abstracts. Of these, 6 were excluded (2 were non-randomized prospective cohort studies with control arms, 4 were conference abstracts only, 2 of which were conference abstracts of full texts already included and 2 of these were conference abstracts that did not have any published full-texts we could find [29, 30].

### Study characteristics

Ten trials, including 1881 participants, met the study eligibility criteria. The median sample size was 98 (interquartile range 72 to 197). Among the five studies that reported follow-up duration, the median was 23 months. All studies were published in English peer-reviewed journals. Table 1 provides the summary characteristics of the identified studies that met inclusion criteria. All studies reported data on OS. Among the 10 trials included, only that of Kawata et al. [21] did not report PFS data. No trial reported baseline cardiovascular characteristics or cardiovascular deaths.

**Table 1:** Characteristics of trials meeting eligibility criteria.

First Author (Last name)	Year of Publication	Country	Cancer Type	Disease Stage	Median Cohort Age	Experimental Arm	Control arm	Concurrent cancer therapy given to both arms	*n* Statin group/Control	Median Follow-up (mo)	Hazard Ratio OS	Hazard Ratio PFS	Median survival statin (mo) with 95% CI	Median survival control (mo) with 95% CI
**Seckl [31]**	2017	United Kingdom	Small-Cell Lung Cancer	Limited or extensive disease	64	Pravastatin 40mg/day	Placebo	Etoposide + cisplatin or carboplatin	422/424	39.6	1.01 (0.88–1.16)	0.98 (0.85–1.13)	10.7	10.6
**Lee [32]**	2017	South Korea	Non-adenocarcinomatous non-small Cell Lung Cancer	Advanced (stage IIIB/IV that progressed after first line chemotherapy)	63	Simvastatin 40mg/day	No placebo	Afatinib 40mg/day	36/32	22.3	0.97 (0.56–1.72)	1.38 (0.84–2.29)	10 (6.4–13.8)	7 (6.1–7.9)
**El-Hamamsy [16]**	2016	Egypt	Any	Brain metastases	55	Simvastatin 80mg/day	No placebo	30-Gy whole brain radiation therapy	25/25	12	0.95 (0.52–1.75)	0.77 (0.43–1.39)	3.4 (0.69–6.01)	3 (2.46–3.54)
**Lim [17]**	2015	South Korea	Colorectal cancer	Metastatic (stage IV)	57	Simvastatin 40mg/daily	Placebo	FOLFIRI/XELIRI	134/135	NR	0.84 (0.64–1.09)	1.03 (0.77–1.37)	15.3 (12.1–18.5)	19.9 (16.8–21.6)
**Kim [33]**	2014	South Korea	Gastric cancer	Metastatic (stage IV)	54	Simvastatin 40mg/daily	Placebo	Capecitabine-cisplatin	120/124	NR	0.97 (0.72–1.29)	0.93 (0.68–1.26)	11.6	11.5
**Hong [18]**	2014	South Korea	Pancreatic cancer	Advanced (metastatic or unresectable)	58	Simvastatin 40mg/daily	Placebo	Gemcitabine	58/56	NR	1.14 (0.77–1.69)	1.08 (0.73–1.60)	6.6 (4.5–8.7)	8.9 (5.3–12.4)
**Hus [19]**	2011	Poland	Multiple Myeloma	Relapsed or refractory	61	Lovastatin 2mg/kg days 1–5 and 8–12 and 0.5mg/kg days 15–28 of each cycle	No placebo	Thalidomide + dexamethasone	49/42	NR	0.68 (0.35–1.32)	0.59 (0.31–1.11)	49	39.5
**Han [34]**	2011	South Korea	Non-small cell lung cancer	Locally advanced or metastatic (stage IIIB-IV)	59	Simvastatin 40mg/daily	No placebo	Gefitinib 250mg/day	52/54	30	0.88 (0.57–1.35)	0.89 (0.60–1.32)	13.6	12
**Konings [20]**	2010	Netherlands	Gastric cancer	Advanced, not amenable to curative resection	58	Pravastatin 40mg/day	No placebo	Epirubicin, cisplatin, capecitabine	15/15	NR	0.57 (0.27–1.22)	0.58 (0.27–1.25)	8 (3.02–12.98)	6 (4.93–7.08)
**Kawata [21]**	2001	Japan	Hepatocellular carcinoma	Advanced (unresectable)	62	Pravastatin 20-40mg/day	No placebo	Transcatheter arterial embolization followed by oral 5-FU for 2 months then randomized to treatment or control	41/42	11	0.61 (0.39–0.96)	.	18	9

Hazard ratio <1 indicates protective effect of statin.

NR: not reported. FOLFIRI: Folinic acid + fluorocuracil + irinotecan. XELIRI: Capecitabine + irinotecan. 5-FU: fluorouracil.

### Cancer survival outcomes

**Fig 2:**
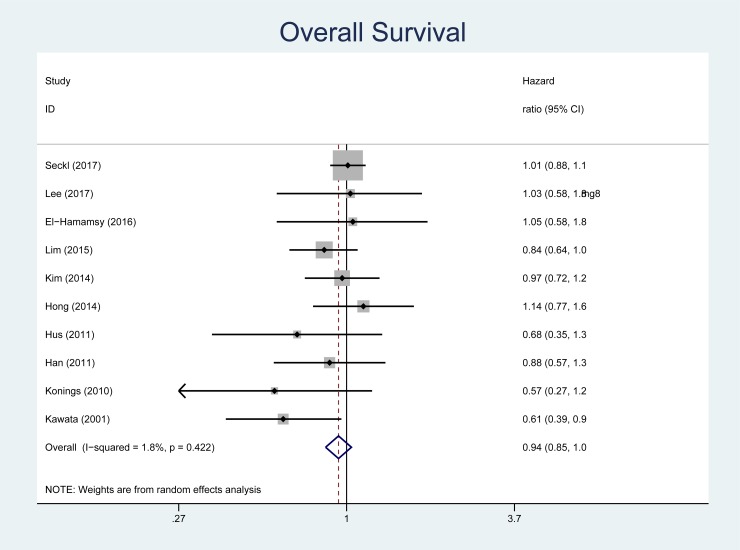
Forest plot for overall survival. The size of the data markers represents the relative weight of the trial according to size and occurrence of the outcome being measured. Hazard ratio <1 indicates benefit with statin.

**Fig 3:**
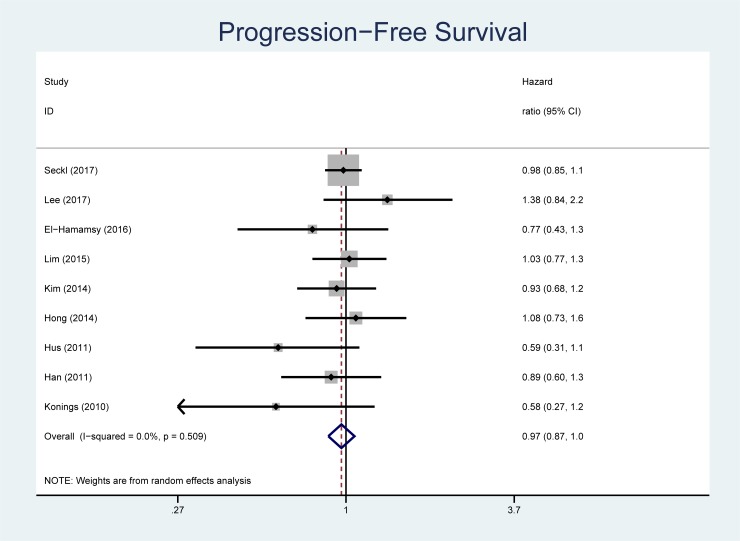
Forest plot for progression-free survival. The size of the data markers represents the relative weight of the trial according to size and occurrence of the outcome being measured. Hazard ratio <1 indicates benefit with statin.

**Fig 4:**
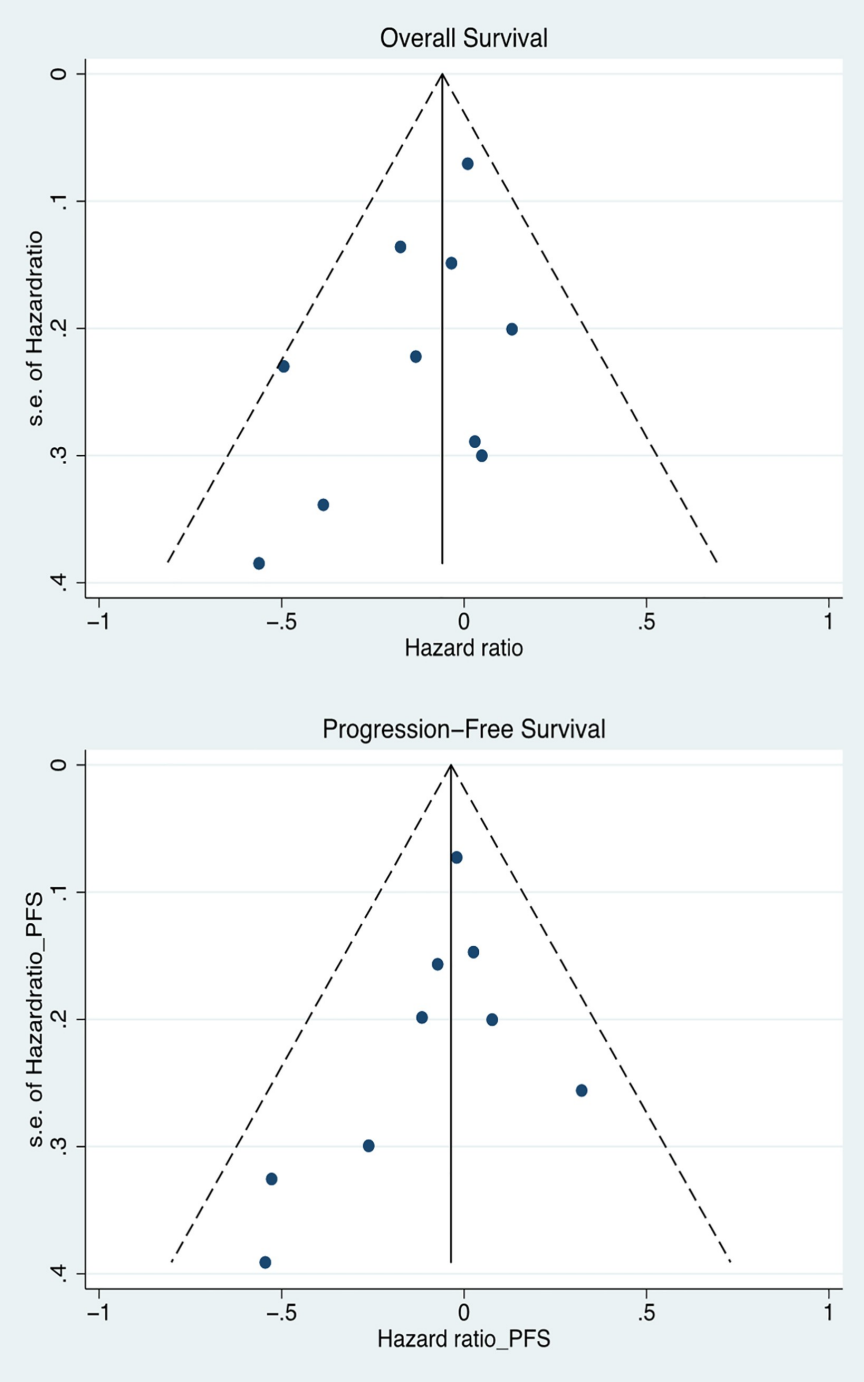
Funnel plots for overall survival and progression-free survival. Plots represent data from 10 studies of OS and 9 studies of PFS.

There were 1,572 deaths among the 1,881 individuals studied. The pooled HR for mortality comparing statin plus standard therapy versus standard therapy alone was 0.94 (95% CI, 0.85 to 1.04, Fig 2). One study did not report PFS [21]. In the 9 studies that reported PFS (n = 1,798 participants), the pooled HR for statin plus standard therapy versus standard therapy alone was 0.97 (95% CI, 0.87 to 1.07, Fig 3). No statistical heterogeneity was observed on analysis of the *I*^*2*^ statistic for either outcome (*I*^*2*^ = 1.8%, p = 0.42 for OS; *I*^*2*^ = 0%, p = 0.51 for PFS). Publication bias was not evident on visual inspection of the funnel plot (Fig 4). We confirmed this finding with the Begg rank correlation (p = 0.92 for OS; p = 0.60 for PFS) and the Egger weighted regression method (p = 0.40 for OS and p = 0.49 for PFS).

Subgroup analysis were performed evaluating hydrophilic statins alone (pravastatin) compared to hydrophobic statins alone (simvastatin and lovastatin). The results did not show a signal towards benefit or harm with specific statin therapy with respect to OS and PFS. Since Seckl et al. accounted for around 50% of the pooled effect for both analyses, we performed a sensitivity analysis excluding this trial. Pooled HR for OS without this trial was 0.87 (95% CI 0.76 to 1.00) and pooled HR for PFS was 0.95 (95% CI 0.82 to 1.10).

## Discussion

Our systematic review and meta-analysis investigated the effect of statin therapy on cancer-survival outcomes in randomized clinical trials of cancer patients. When added to conventional chemotherapy, statins did not improve overall survival or progression-free survival. This is at odds with preclinical data that suggest statins have anti-tumor effects and our results contrast with a number of observational studies showing statin use to be associated with improved cancer-specific outcomes and all-cause mortality.

### Explaining the divergence between laboratory and clinical trial findings on the anti-cancer effects of statins

Theoretically, statins may exert anti-tumor effect by inhibiting 3-hydroxy-3-methyl-glutaryl-coenzyme A (HMG-CoA) reductase, the rate limiting step mevalonate synthesis. This inhibition affects downstream products of mevalonate, which regulates cell membrane integrity, cell signaling, protein synthesis and cell cycle progression [6]. There is evidence to suggest that this effect of statins on tumor growth and angiogenesis may be dose related. Statins have been shown to be pro-angiogenic at low concentrations (0.5mg/kg/day of cerivastatin and atorvastatin) and anti-angiogenic at higher concentrations (2.5mg/kg/day) [35]. Although low dose statins may not alter tumor growth or angiogenesis, they have been theorized to increase the sensitivity of the cancer cell to chemotherapy by affect signalling pathways such as Ras [7]. This may explain why most of the clinical trials have favored continuous, low-dose statin regimens over brief, high-dose statin regimens, with the exception of Hus et al. Furthermore, low-dose regimens are associated with a lower risk for statin side effects, including hepatotoxicity, myopathy and rhabdomyolysis. Since cancer patients often receive concomitant medications, they are more likely to develop statin-related side effects, as these medications may interfere with statin metabolism through the cytochrome P-450 system [5, 36]. We also performed a subgroup analysis comparing hydrophilic statins (pravastatin) with hydrophobic statins (lovastatin and simvastatin), to determine if differences in hydrophilicity and pharmacokinetic properties have clinical significance. However, our results showed no advantage of either type of statin compared to control with respect to our primary and secondary outcomes. Finally, some statins may be more potent than others. Cerivastatin has been shown to be 10 times more potent than atorvastatin and lovastatin in inducing apoptosis in acute myeloid leukemia cell lines [37], although it is unknown why cerivastatin has such a profound effect, as all three are lipophilic statins. Thus, it is possible that the dose or type of statin used in clinical trials may be suboptimal with respect to impacting on cancer outcomes.

### Explaining the divergence between observational data and randomized clinical trial findings on the anti-cancer effects of statins

In individuals from the general population at moderate or high risk of cardiovascular disease, there is abundant randomized, controlled clinical trial evidence of the beneficial effects of statins on cardiovascular disease. It is uncertain whether patients with cancer derive the same cardiovascular benefit from statins as non-cancer population; there is equipoise because cancer patients may die from their cancer before they can derive a cardiovascular benefit from statins. The cancer patients included in this meta-analysis had a mean median overall survival of 14.6 months, suggesting that these patients generally had a poor prognosis. The prognosis in these patients was largely driven by their cancer, which is a major competing risk for cardiovascular disease. No trial reported cardiovascular outcomes. In contrast, observational data of statin use and outcomes in cancer patients are typically from registries that record longer follow-up, and generally include patients with less-advanced cancer, resulting in a selection bias. For example, the observational data meta-analysis of 1,111,407 cancer patients by Mei et al. reported a median follow-up of 50 months, compared to a median follow-up of 23 months in this meta-analysis, and only 7 out of the 95 studies analysed included patients with only metastatic disease [13]. Cardiovascular benefit from statins can be seen as early as within 1-year of use, but the effect is greatest after 3–4 years of therapy. It is possible that the mortality benefit seen in observational data is from the cardiovascular benefit derived from statins, rather than an anti-cancer effect [14].

In addition to selection bias, observational studies are also subject to additional biases such as immortal time bias, reporting bias, and misclassification bias. Immortal time bias refers to a span of time in the observation or follow-up period of a cohort during which the outcome under study (in our case, death) could not have occurred. It usually occurs with the passing of time before a subject initiates a given exposure. While a subject is not truly immortal during this time span, the subject necessarily had to remain event free until the start of exposure to be classified as exposed, which biases the results in favour of the treatment under study by conferring a spurious survival advantage to the treated group [38]. Adherence to statin therapy is low in a non-randomized trial setting, resulting in reporting bias. In a Canadian study of 85,020 patients in whom statin therapy was prescribed for primary prevention, only 25% of patients continued taking the medication at 2-year follow-up. [39]. Misclassification bias is also common in observational studies and the differentiation between “statin users” and “non-statin users” may not be not as rigorous as in randomized trials. Although the last two biases may result in the dilution of treatment effect in observational studies, the other biases mentioned and potential for the presence of unmeasured confounders due to the lack of randomization can result in spurious associations made when interpreting observational data, as in the case of hormone replacement therapy and cardiovascular disease.

There are some limitations to this meta-analysis. First, this is a meta-analysis of aggregate patient data, rather than individual patient data. Individual patient data meta-analyses allow for harmonisation of inclusion and exclusion criteria, follow-up times, confounding co-variates and variable definitions. However, there is typically good concordance between summary data meta-analyses and individual patient data meta-analyses and in our study, the definition of our primary outcome, overall survival, remained constant across all studies [40]. Second, this meta-analysis includes patients with a heterogenous group of cancers. Some of these cancers are associated with a better prognosis (stage IIIB non-small cell lung cancer, limited stage small cell lung cancer) and others with brain metastases and a median survival of 3 months. Although including a variety of cancers introduces clinical heterogeneity to our analysis, the putative anti-cancer activity of statins is mostly through the mevalonate pathway that is common to the cancers studied in this analysis, and therefore, the effects of statins should be seen independent of cancer type. The small sample sizes of most of the included trials does not rule out the possibility that statins may have anti-cancer effect in certain subgroups. For example, Han et al. reported a higher response rate and longer PFS in patients with wild-type EGFR non-adenocarcinomatous non-small cell lung cancers who were given simvastatin [34]. Third, some trials are placebo controlled and others are not, although there was no statistical heterogeneity and the calculated *I*^2^ was 0. Fourth, a single study (Seckl et al. [31]) accounted for nearly 50% of the pooled effect in the analysis of OS and greater than 50% of the effect in the analysis of PFS. However, a sensitivity analysis performed excluding this trial revealed similar results. Furthermore, this was a large, well-conducted RCT with no limitations that limit the validity of their results. Finally, the hazard ratio and 95% CI for six trials were not directly reported and were instead estimated by inspection of their Kaplan-Meier curves using the methods of Tierney and Parmar which make assumptions on the number of patients censored. Despite those limitations, this study has its strengths. This is the first meta-analysis of randomized controlled trials of statin therapy in addition to usual anti-cancer therapy in patients with active cancer. Therefore, our conclusions are not subject to the biases described in observational data. We used hard endpoints of overall survival and progression-free survival. We estimate that the 1,881 patients studied, 1,572 (84%) of whom died, should confer >90% power to detect a hazard ratio of as low as 0.8. This suggests that in the populations studied in this meta-analysis, lack of power is very unlikely to account for the neutral treatment effect of statins. Finally, we used a comprehensive search strategy and our included studies were subject to minimal publication bias and between-study heterogeneity.

To conclude, in patients with cancer and a prognosis <2 years, statins do not appear to improve overall survival or progression-free survival. More research is needed to evaluate the effect of high-dose statin therapy in cancer patients and research is needed in cancer patients with a better prognosis (e.g. prostate cancer) to determine whether they achieve an anticancer or cardiovascular benefit from longer-term statin therapy.

## Supporting information

S1 File Search strategy. (PDF)

S2 File PRISMA checklist. (DOC)

S1 Table Risk of bias assessment. (DOCX)
