# Supplementary material for: Statin therapy in the treatment of active cancer: A systematic review and meta-analysis of randomized controlled trials
Source: PLoS One. 2018 Dec 20;13(12):e0209486. doi: 10.1371/journal.pone.0209486 (PMC6301687; doi:10.1371/journal.pone.0209486)
Supplement: S1 File — (PDF) [file pone.0209486.s001.pdf]

Database: OVID Medline Epub Ahead of Print, In-Process & Other Non-Indexed Citations, Ovid MEDLINE(R) Daily and Ovid MEDLINE(R) 1946 to Present
Search Strategy:
----
1 exp Neoplasms/ (3027278)
2 neoplas*.ti,ab,kf. (366529)
3 cancer*.ti,ab,kf. (1511459)
4 malignan*.ti,ab,kf. (507516)
5 tumo?r*.ti,ab,kf. (1497713)
6 carcino*.ti,ab,kf. (724847)
7 adenocarcinoma*.ti,ab,kf. (128964)
8 sarcoma*.ti,ab,kf. (92210)
9 adenoma*.ti,ab,kf. (78517)
10 chondrosarcoma*.ti,ab,kf. (7382)
11 fibrosarcoma*.ti,ab,kf. (11261)
12 dermatofibrosarcoma*.ti,ab,kf. (1758)
13 neurofibrosarcoma*.ti,ab,kf. (402)
14 hemangiosarcoma*.ti,ab,kf. (1002)
15 leiomyosarcoma*.ti,ab,kf. (9566)
16 liposarcoma*.ti,ab,kf. (5808)
17 myosarcoma*.ti,ab,kf. (231)
18 rhabdomyosarcoma*.ti,ab,kf. (11066)
19 myxosarcoma*.ti,ab,kf. (248)
20 osteosarcoma*.ti,ab,kf. (20383)
21 lymphoma*.ti,ab,kf. (161293)
22 or/1-21 (3910941)
23 exp Hydroxymethylglutaryl-CoA Reductase Inhibitors/ (35923)
24 hmg coa.ti,ab,kf. (8109)
25 hydroxymethylglutaryl coa.ti,ab,kf. (641)
26 hydroxymethylglutaryl-coenzyme.ti,ab,kf. (618)
27 (statin or statins).ti,ab,kf. (33545)
28 atorvastatin*.ti,ab,kf. (7486)
29 fluvastatin.ti,ab,kf. (1736)
30 lovastatin.ti,ab,kf. (3576)
31 pitavastatin.ti,ab,kf. (752)
32 pravastatin*.ti,ab,kf. (3856)
33 rosuvastatin*.ti,ab,kf. (2937)
34 simvastatin*.ti,ab,kf. (8502)
35 or/23-34 (56163)
36 random*.mp. (1183355)
37 RCT*.mp. (35972)
38 exp clinical trial/ or randomized controlled trial/ (792176)
39 Random Allocation/ (93718)
40 exp clinical trials as topic/ or randomized controlled trials as topic/ (312133)
41 double-blind method/ or single-blind method/ (168967)
42 ((single or double or triple or treble) adj2 (blind* or mask*)).ti,ab,kf. (156401)
43 randomized controlled trial.pt. (457171)
44 "clinical trial*".mp. (922729)
45 or/36-44 (1767745)
46 22 and 35 and 45 (1140)
47 animals/ not (humans/ and animals/) (4407637)
48 46 not 47 (1068)

****
