# Supplementary material for: Statin therapy in the treatment of active cancer: A systematic review and meta-analysis of randomized controlled trials
Source: PLoS One. 2018 Dec 20;13(12):e0209486. doi: 10.1371/journal.pone.0209486 (PMC6301687; doi:10.1371/journal.pone.0209486)
Supplement: S1 Table — (DOCX) [file pone.0209486.s003.docx]

|  | Selection bias | | Performance bias | Detection bias | | Attrition bias | | Reporting bias |
| --- | --- | --- | --- | --- | --- | --- | --- | --- |
| Study, Year | **Random sequence generation** | **Allocation concealment** | **Blinding of participants and personnel** | **Blinding of outcome assessment (mortality)** | **Blinding of outcome assessment (progression events)** | **Incomplete outcome data (mortality)** | **Incomplete outcome data (progression events)** | **Selective reporting** |
| Seckl, 2017 | Used minimization | Unclear | Researchers and patients | N/A | Unclear | Similar attrition in both groups | Unclear | Protocol not available, but the study reports all expected outcomes |
| Lee, 2017 | Unclear | Unclear | Did not mention if the study was blinded | N/A | Unclear | Similar attrition in both groups | Unclear | Protocol not available, but the study reports all expected outcomes |
| El-Hamamsy, 2016 | Unclear | Unclear | Did not mention if the study was blinded | N/A | Unclear | Similar attrition in both groups | Unclear | Protocol not available, but the study reports all expected outcomes |
| Lim, 2015 | Used minimization | Unclear | Researchers and patients | N/A | Unclear | Similar attrition in both groups | Unclear | Protocol not available, but the study reports all expected outcomes |
| Kim, 2014 | Unclear | Unclear | Researchers and patients | N/A | Unclear | Similar attrition in both groups | Unclear | Protocol not available, but the study reports all expected outcomes |
| Hong, 2014 | Used minimization | Unclear | Researchers and patients | N/A | Unclear | Unclear | Unclear | Protocol available, expected outcomes reported |
| Hus, 2011 | Unclear | Unclear | Did not mention if the study was blinded | N/A | Unclear | Unclear | Unclear | Protocol not available, but the study reports all expected outcomes |
| Han, 2011 | Unclear | Unclear | Open label | N/A | Unclear | Similar attrition in both groups | Unclear | Protocol not available, but the study reports all expected outcomes |
| Konings, 2010 | Unclear | Unclear | Did not mention if the study was blinded | N/A | Unclear | Unclear | Unclear | Protocol not available, but the study reports all expected outcomes |
| Kawata, 2001 | Computer program | Unclear | Did not mention if the study was blinded | N/A | Unclear | Similar attrition in both groups | Unclear | Protocol not available, but the study reports all expected outcomes |
